# Submitted for Your Consideration: Potential Advantages of a Novel Clinical Trial Design and Initial Patient Reaction

**DOI:** 10.3389/fgene.2012.00145

**Published:** 2012-08-08

**Authors:** Matthew Shane Loop, Alexis C. Frazier-Wood, Amy S. Thomas, Emily J. Dhurandhar, James M. Shikany, Gary L. Gadbury, David B. Allison

**Affiliations:** ^1^Section on Statistical Genetics, Department of Biostatistics, Ryals School of Public Health, University of Alabama at BirminghamBirmingham, AL, USA; ^2^Department of Epidemiology, Ryals School of Public Health, University of Alabama at BirminghamBirmingham, AL, USA; ^3^Division of Preventive Medicine, School of Medicine, University of Alabama at BirminghamBirmingham, AL, USA; ^4^Nutrition Obesity Research Center, University of Alabama at BirminghamBirmingham, AL, USA; ^5^Office of Energetics, Ryals School of Public Health, University of Alabama at BirminghamBirmingham, AL, USA; ^6^Department of Statistics, Kansas State UniversityManhattan, KS, USA

**Keywords:** treatment response heterogeneity, crossover design, Balaam design

## Abstract

In many circumstances, individuals do not respond identically to the same treatment. This phenomenon, which is called treatment response heterogeneity (TRH), appears to be present in treatments for many conditions, including obesity. Estimating the total amount of TRH, predicting an individual’s response, and identifying the mediators of TRH are of interest to biomedical researchers. Clinical investigators and physicians commonly postulate that some of these mediators could be genetic. Current designs can estimate TRH as a function of specific, measurable observed factors; however, they cannot estimate the total amount of TRH, nor provide reliable estimates of individual persons’ responses. We propose a new repeated randomizations design (RRD), which can be conceived as a generalization of the Balaam design, that would allow estimates of that variability and facilitate estimation of the total amount of TRH, prediction of an individual’s response, and identification of the mediators of TRH. In a pilot study, we asked 118 subjects entering a weight loss trial for their opinion of the RRD, and they stated a preference for the RRD over the conventional two-arm parallel groups design. Research is needed as to how the RRD will work in practice and its relative statistical properties, and we invite dialog about it.

## Introduction

Due to the varied environmental, genetic, and physiological milieu from person to person, a given treatment does not produce the same response in all patients. Weight loss treatments for obesity and its related comorbidities are no exception, and a wide range of weight loss and metabolic changes occurs with most treatments (Bouchard et al., 1990, 1994; Bray, 2008; Puzziferri et al., 2008). While variability in response to a given treatment occurs among persons in many clinical conditions, we shall use obesity as an example to illustrate the effects of this phenomenon for purposes of exposition. Obesity, like many chronic conditions the medical community faces, is a complex condition likely to have many causes and many solutions (McAllister et al., 2009). Certain genes are established contributors to phenotypic variation in body mass index (BMI; Wang et al., 2012), and it is possible that genotypic variation could also contribute to variation in response to a weight loss treatment. Quantifying variation in treatment response and identifying “responders” and “non-responders” would improve treatment allocation for any complex disease, and is imperative for optimizing obesity treatment for individuals in a heterogeneous population. In addition, quantifying variation due to patient-treatment interaction is of interest to all investigators seeking to identify effective obesity treatments, as it is a source of variation that, if identified, could lead to a clearer picture of a treatment’s effect among persons.

Several approaches are currently used to examine heterogeneity in treatment response. Association between degree of change in the outcome variable from baseline with other baseline covariates is often used to identify predictors of change in the outcome variable (Sysko et al., 2010; Guaraldi et al., 2011). In addition, genome wide association studies (GWAS) are used to estimate the amount of inter-individual variability in weight loss that is due to genetic differences (Sarzynski et al., 2011), and behavioral compensation (e.g., increased energy intake or decreased non-exercise energy expenditure) has been proposed as an explanation for among person variability in weight loss when subjects lose less weight than predicted (Manthou et al., 2010; Turner et al., 2010). Covariates such as genotype, age, starting weight, race, or gender can explain a portion of the inter-individual variability in weight loss trials, but we are left knowing neither the magnitude of the total inter-individual variability nor the proportion of inter-individual variability that those covariates explain.

**Figure 1:**
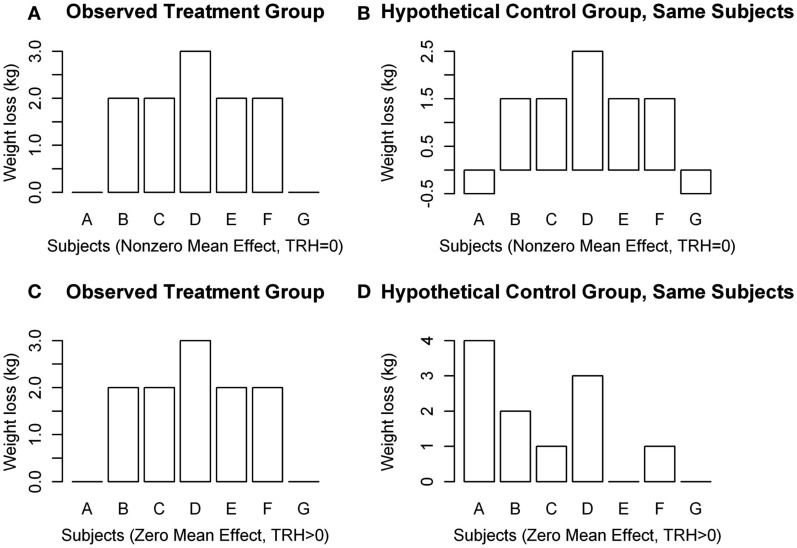
**Hypothetical distributions of individual weight changes in a clinical trial. (A,B)** Show μ*_D_* ≠ 0, TRH = 0, and **(C,D)** show μ*_D_* = 0, TRH > 0.

There is a gap, however, between standard methods for assessing a treatment’s efficacy and our desire to characterize this inter-individual variability in treatment response, which is called treatment response heterogeneity (TRH). Methods currently employed do not allow for quantification of individual treatment *response*, which is defined as the difference between the change in the outcome variable following treatment compared to change in the *same* outcome variable if the *same* individual had received the control treatment in the *same* period of time (Gadbury, 2010). A common mistake made when discussing treatment effect is labeling an observed *change* as a *response*. For instance, let us consider Figure 1, which is an illustration of potential situations that could occur in a two-arm parallel groups randomized controlled trial (RCT). *D* refers to the difference between weight loss on treatment and weight loss on control, or *D* = *T* − *C*, μ*_D_* is the population mean of *D*, and TRH refers to the variance in *D* that is due to subject-treatment interactions. Subjects A and G in Figure 1A would often be labeled “non-responders” if a standard two groups design were used in the RCT. As is revealed in Figure 1B, however, both subjects would have gained 0.5 kg on the control, making the treatment effect for subjects A and G 0.5 kg lost, just like all of the other subjects. Even though no change is observed for subjects A and G in the treatment group, the effect of the treatment on these subjects is not necessarily zero. Therefore, the label “non-responder” is unjustified if derived in a standard design. Figures 1C,D both show a mean weight loss of 1.57 kg, indicating an average treatment effect of 0 kg lost. However, the treatment effect was non-zero for subjects A, C, E, and F.

**Figure 2:**
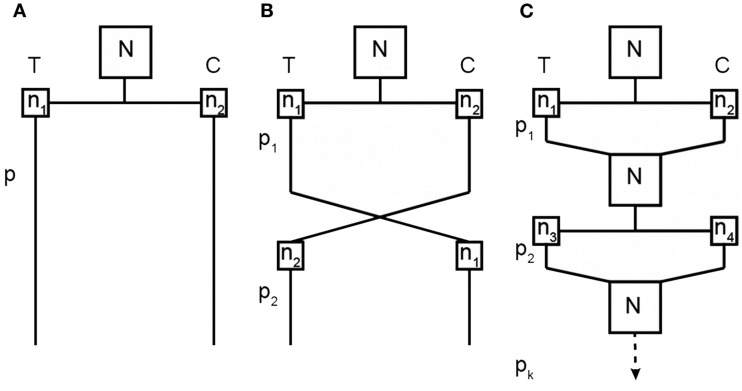
**Comparison of a two-arm parallel groups design (A), a two-period crossover design (B), and the RRD (C)**. T represents the treatment group and C the control group; *N* is the total sample size for each experiment; *n* is the group size for a particular treatment condition at a particular time point (with subscripts differentiating between unique groups of subjects); and *p* is the treatment period (with subscripts identifying which treatment period).

Figure 1 illustrates several points concerning TRH: (1) It is possible that μ*_D_* ≠ 0, but TRH = 0 (Figures 1A,B); (2) it is possible that μ*_D_* = 0, but TRH > 0 (Figures 1C,D); (3) variability in weight loss among subjects in the treatment group does not, by itself, indicate the existence of TRH; and (4) subjects who lose the least amount of weight do not necessarily have a weaker response to the treatment. Observing individual changes provides an estimate for mean population response, but no statements can be made about individual responses in a conventional design.

Of the RCT designs that might be considered when discussing estimation of variance due to subject-treatment interactions, the two most well known are the two-period crossover design and the Balaam design. In the two-period crossover design, subjects are randomly assigned to one of two possible sequences of treatment A and treatment B (or placebo): AB or BA. The two-period crossover design cannot separate the inter-individual variability in treatment response from other sources of variability (Senn, 2001). Tucker-Drob (2011) suggested adding a third sequence, AA, to further distinguish between treatment effects and other sources of variability. However, this approach does not allocate multiple periods of each treatment to each individual, and thus it has limitations for estimating subject-treatment interaction (Senn, 2001).

The Balaam design modifies the two-period crossover by increasing the possible number of sequences that patients can be randomized to: AA, AB, BA, or BB (Balaam, 1968). An advantage of the Balaam design is that the effect of time-treatment interactions can be accounted for. However, because participants in a Balaam design do not experience multiple periods of all treatments, estimates of TRH do not separate the variability due to individual patient-treatment interaction from other sources of variability (much like the two-period crossover design and the design proposed by Tucker-Drob, 2011) (Senn, 2001). Additionally, we can see that because only half the subjects receive both treatments (in a balanced design), one could provide reasonable estimates for the individual mean effect of treatment, as well as the sample variance of those means, for only half of the patients in the study sample (Balaam, 1968). The Balaam design enhances the picture of how variable treatment effects are in a population, but it does not uniquely estimate patient-treatment interactions or the variance thereof.

To bridge the gap between our current methods of assessing treatment effects and the desired knowledge of inter-individual variability in treatment response, we propose a novel RCT design involving repeated randomizations of each subject to treatment or control, which we refer to as the repeated randomizations design (RRD). Figure 2 provides a diagram that compares the two-arm parallel groups design (PGD), the two-period crossover design, and the RRD. The Balaam design can be thought of as a special case of the RRD with only two treatment periods. Alternatively, the RRD can be thought of as a randomized form of aggregated *n*-of-1 trials, the non-randomized form having been discussed elsewhere (Franklin et al., 1996; Zucker et al., 1997; Nikles et al., 2011).

Because of the nature of the randomization, which repeatedly switches subjects between two treatments (or between a treatment and a placebo), there are important practical restrictions that dictate which interventions/conditions are appropriate for the RRD. Two main characteristics influence whether an RRD design is appropriate: (1) an individual can be easily switched from one experimental intervention to the other; and (2) an individual can be *ethically* switched from one experimental intervention to the other. Minimal carry-over effects of the treatment, as well as the ability to blind the treatment, are characteristics that are facilitative but not critical. Clinical interventions for weight loss that would often fit with these characteristics include, but are not limited to: pharmaceuticals, dietary supplements and other dietary interventions, and exercise interventions. In contrast, gastric bypass surgery would generally not be appropriate to test using the RRD. The experimental intervention being tested is not restricted to clinical interventions, but could test an aspect of metabolism, behavior, or weight change that is of scientific interest, and satisfies the characteristics laid out above (e.g., short-term metabolic effects of different macronutrients).

The conduct of a trial using an RRD would proceed as follows. *N* subjects would be randomized at baseline to either treatment or control. After a pre-specified follow-up period, the outcome of interest (e.g., weight change) would be measured on all subjects. Subjects would then be randomized again to either treatment or control. This process would continue for a total of *p* treatment periods. With a large enough *p*, the probability of one subject receiving only treatment (or only control) throughout the trial is effectively zero. Specifically, the probability of receiving only treatment or only control is ${\left(\frac{1}{2}\right)}^{p}+{\left(\frac{1}{2}\right)}^{p}=2{\left(\frac{1}{2}\right)}^{p}={\left(\frac{1}{2}\right)}^{p-1}$. Therefore, the authors suspect that participants would prefer such a design compared to the classic PGD, in which an individual subject has a 50% chance of receiving only control (in a balanced design). With *p*_T_ number of observations on treatment and *p*_C_ number of observations on control, the following estimates could be computed, among others: (1) the sample estimate of the mean effect of the treatment for an individual; (2) the sample estimate of the variance of all individual mean effects of the treatment; and (3) the sample estimate of the mean effect of the treatment for the population. Estimation of the total amount of TRH would also facilitate estimates of the proportion of inter-individual variability in treatment response a covariate of interest might explain (e.g., genotype).

These multiple observations on *each* treatment for *each* subject allow for more direct evaluation of individual subject-treatment interactions (Senn, 2001). From this study design, investigators will have not only an estimate for the mean effect of a treatment within a population, but also, with making some relatively mild and plausible assumptions (e.g., the non-estimable correlation between the two treatment outcome variables is the same across periods): (1) an estimate of the total inter-individual variability in treatment response; (2) the proportion of true non-responders; and (3) the proportion of the population for whom a standard treatment works better than an experimental treatment, even though the experimental treatment appears to be better on average. This design would thereby provide information about individual responses, not just the population’s mean response.

Because repeated randomization to different treatment groups would greatly alter the study experience for participants, however, we conducted a survey to determine how willing participants would be to enroll in such a study.

## Materials and Methods

We ascertained how acceptable participating in such a design would be by conducting a survey of subjects being screened for, or already enrolled in, a weight loss trial investigating a diet intervention (the *Medifast 5 & 1 Plan*). After obtaining approval from the Institutional Review Board of the University of Alabama at Birmingham, the protocol for the trial was modified to include a printed questionnaire (The Clinical Trials.gov identifier NCT01211301). Written informed consent was obtained from all subjects. Since the survey was added after recruitment began, it was administered to subjects at one of two time points: screening or follow-up. Some subjects screened for the trial never took the survey because they had already been screened out, or they were lost to follow-up after randomization.

Subjects (*n* = 119) were given a description of two potential trial designs (see Table 1), and 118 completed the survey. Trial A described the standard PGD (similar to that used in the trial for which they were being screened), and trial B described the RRD. The five-question instrument investigated which design subjects preferred, in which design subjects believed they were more likely to enroll, and which design they believed they would be more likely to complete. The scales for questions 2 through 5 were 1–5. When the survey was administered to participants, the scale for questions 2 and 3 used 1 as the most negative response and 5 as the most positive response. Questions 4 and 5 were reverse scored so that 1 was the most positive response and 5 was the most negative response. This reversal was done to decrease the effect of acquiescence (Cloud and Vaughan, 1970). In the analyses, the responses to question 4 were flipped to the other side of the scale (e.g., a response of “4” became a response of “2”). The same process was done for the responses to question 5.

**Table 1:** **Description of designs given to study participants**.

Design	Description
Study A – single randomization design	In study A, the classic design, if you were to participate you would be randomly assigned to take either the active drug being studied or to a pill with no drug (a placebo). There would be a 50% chance that you will be assigned to take the placebo. The study would be run “double blind” so that neither you nor the study staff would know whether you are taking the active drug or the placebo until after the study is over. The study will proceed like this for 24 months (2 years), and every month you would be asked to come to the clinic and be weighed.
Study B – repeated randomization design	In study B, a new design, if you were to participate, you would be randomly assigned each month to the active drug being studied or to a placebo. You would be assigned to the active drug or placebo an equal number of times; that is, you would be guaranteed to get the drug for 12 of the 24 months in random order. The study will be run “double blind” so that neither you nor the study staff will know whether you are taking the active drug or the placebo during any particular month. The study will proceed like this for 24 months (2 years), and every month you would be asked to come to the clinic and be weighed.

Summary statistics were estimated for all five questions and the available demographic variables. Two null hypotheses were tested. The first null hypothesis was that participants believed they were equally likely to enroll in a trial using the PGD as a trial using the RRD. The second null hypothesis was that participants believed they were equally likely to complete a 2-year trial using the PGD as a 2-year trial using the RRD. To test these two null hypotheses, we used paired Wilcoxon signed rank tests with α = 0.05. Paired tests were used because all participants answered all questions, so a comparison of responses to questions two and three (hypothesis one) and questions four and five (hypothesis two) involved paired data. Non-parametric rank based methods were used as a natural choice for ordinal scale data such as these (Gardner and Martin, 2007).

## Results

Age in the study sample ranged from 20 to 63  years, with a mean (SD) of 40.9 (9.7) years. The ethnic groups represented were White (24.7%), Black/non-Hispanic (73.3%), Black/Hispanic (0.9%), and Asian (0.9%). Females comprised 87.2% of the study sample.

Descriptive statistics of the responses are provided in Table 2. When given a choice between the PGD and the RRD (question 1), 63.6% (95% CI: 54.9, 72.2) preferred the RRD. A paired Wilcoxon signed rank test comparing question 2 and question 3 revealed that subjects believed they were significantly more likely to enroll in a trial using the RRD (*S* = −269.5, *p* = 0.04). However, there was no significant difference in subjects’ stated beliefs about how likely there were to complete a 2-year trial using one design instead of the other (*S* = −77, *p* = 0.31).

**Table 2:** **Survey questions, potential responses, and summary statistics of responses**.

Question	Potential responses*					Mean (95% CI)	Median	Range (min, max)
2. If only design A were available to you, how likely would you be to enroll?	1	2	3	4	5	3.9 (3.7, 4.1)	4	4 (1, 5)
3. If only design B were available to you, how likely would you be to enroll?	1	2	3	4	5	4.1 (3.9, 4.3)	4	4 (1, 5)
4. If you enrolled in a trial of design A, and it was a 2 year trial, how likely would you be to complete the trial?	1	2	3	4	5	3.9 (3.7, 4.1)	4	4 (1, 5)
5. If you enrolled in a trial of design B, and it was a 2 year trial, how likely would you be to complete the trial?	1	2	3	4	5	4.0 (3.8, 4.2)	4	4 (1, 5)

*1 is the most negative response, and 5 is the most positive response. 3 is a neutral response.

## Discussion

We have reported results of a questionnaire from a pilot study that indicates future participants in weight loss trials might prefer to participate in a trial using an RRD to a trial using a PGD. This finding gives some indication that further development of the RRD could be worthwhile. Because the questionnaire was a pilot study, the questionnaire did not undergo rigorous psychometric evaluation. However, we believe that the descriptions of the two trials were clear, and that the questions themselves were understandable to the average participant in a weight loss trial. Additionally, participants in our study were enrolled in a clinical trial testing a dietary intervention, not a pharmaceutical intervention, and it is unknown whether participant opinions of the RRD in a trial testing a pharmaceutical would be different.

As stated previously, the RRD would be inappropriate for evaluating some types of treatments, including pharmaceuticals, as well as for some types of conditions. Pharmaceuticals, for example, that have considerable carry-over effects might not be well-suited for study with the RRD. Additionally, if the condition under study were immediately dangerous or life-threatening, then rapidly switching a participant on and off treatment might not be ethical.

Clinical interventions are believed to produce varied results among persons (e.g., a treatment may produce a different magnitude or direction of response in individuals with different genotypes), but current RCT designs cannot estimate the extent of this inter-individual variability in response. We have proposed an alternative design, which would allow for estimation of the total inter-individual variability in treatment response. Furthermore, evidence from a pilot survey suggests subjects might prefer the RRD to the conventional PGD. We conclude with several topics for future research related to optimizing the use of the RRD:

1. What are the most appropriate analytical procedures for estimating the quantities of interest (i.e., the proportion of the population with negative responses, the proportion of true non-responders, and the proportion of the population that genuinely responds better to a treatment that is inferior to an alternative treatment, on average)?
  Linear mixed models would likely be an appropriate method to use, since they allow for modeling at the individual level (Mallinckrodt et al., 2003). Additional topics concerning the analysis procedure, such as the handling of missing data, could be addressed using established methods (e.g., multiple imputation; Elobeid et al., 2009). Research comparing such analytic approaches in RRDs would be warranted.
2. What is the relative efficiency of the RRD compared with the PGD for estimating interactions between treatment effects and measured covariates (e.g., genotype)? What is the relative efficiency of the RRD compared with the PGD for estimating mean treatment effects?
  The relative efficiency will likely depend upon the degree of residual dependence across time, within individuals, as well as other factors. Deriving analytic expressions of the relative efficiency under varying circumstances would help investigators to choose between RRDs and PGDs when such questions are of interest.
3. What are the advantages and disadvantages of constraining the randomizations so that each subject receives an equal allocation of treatment and control periods?
  At a social level, there are marked advantages to such constraining because completely randomized allocation of treatment periods in a long-term trial, in which a placebo is the comparison treatment, can make some subjects hesitant to participate (AD2000 Collaborative Group, 2004). Each subject will then know that they will receive active treatment at least half of the time. Additionally, equal sample sizes within an individual (i.e., equal number of treatment and control periods) would allow for greater precision in estimating an effect of treatment for a given individual. A disadvantage is that if the study is not completely blinded, at some point a patient’s next treatment condition will be predictable.
4. How would the RRD actually affect subject recruitment and retention?
  Our study reported how likely one group of subjects *believed* they would be to complete a trial using the RRD. It is well known that drop-out rates in obesity trials can be quite large (Elobeid et al., 2009). The actual effect of the RRD on subject retention cannot be known until a RRD is used.

### Application to pharmacogenetics

Since the RRD could estimate the total inter-individual variability in treatment response, it could help determine the potential impact of subsequent analyses attempting to explain that variability. Standard methods of estimating the proportion of variability in a phenotype attributable to genetics exist (e.g., GWAS). These methods can be expensive. Therefore, knowing when the probability for a “return on the investment” is small (i.e., when the total TRH is small) might be helpful to both investigators and funding bodies. To our knowledge, no other RCT designs can provide estimates of total TRH as reliable as the RRD.

We hope this work will spark dialog within the scientific community regarding estimation of inter-individual variability in treatment response, as well as the feasibility of the RRD.

## Conflict of Interest Statement

David B. Allison, Ph.D. has served as a consultant to Medifast. This does not alter our adherence to all the Frontiers policies on sharing data and materials. Dr. Allison has, anticipates, or has had financial interests with the Frontiers Foundation; the Federal Trade Commission, Vivus, Inc.; Kraft Foods; University of Wisconsin; University of Arizona; Paul, Weiss, Wharton, and Garrison LLP; and Sage Publications.
